# Preliminary study on the participation of TLR9 on erythrocyte surface combined with mtDNA in the monitoring of infectious diseases

**DOI:** 10.3389/fmed.2024.1498627

**Published:** 2025-02-19

**Authors:** Ling Xiao, Haixia Xu, Siwei Liu, Zhanrui Cheng, Yujie Kong, Li Tian

**Affiliations:** ^1^Department of Blood Transfusion, Deyang People's Hospital, Deyang, China; ^2^Institute of Blood Transfusion, Chinese Academy of Medical Sciences and Peking Union Medical College, Chengdu, China; ^3^Key Laboratory of Transfusion Adverse Reactions, Chinese Academy of Medical Sciences, Chengdu, China; ^4^Department of Laboratory, The First Affiliated Hospital of Chengdu Medical College, Chengdu, China; ^5^School of Clinical Medicine, Chengdu Medical College, Chengdu, China

**Keywords:** red blood cells, infection, toll-like receptor 9, mitochondrial DNA, monitor disease

## Abstract

**Background:**

TLR9 is typically found within cells and plays a crucial role in identifying pathogenic and self-DNA in chronic inflammation and immune complexes. Recent discoveries indicate its presence on the surface of human red blood cells, where it engages in immune regulation by binding to free mtDNA. The purpose of this study is to explore the role of TLR9 as a pattern recognition receptor combined with mtDNA in the monitoring of infectious diseases.

**Methods:**

TLR9 presence on the surface of red blood cells was assessed using flow cytometry in both healthy individuals and patients with bacterial infections. Subsequently, DNA bound to the red blood cell surface was extracted separately from both groups. The absolute quantification of mtDNA copy numbers within the extracted DNA was conducted using qPCR technology, followed by statistical analysis. Additionally, the correlation between mtDNA copy numbers bound to red blood cell surfaces in bacterial infection patients with varying CRP concentrations was examined using univariate linear regression.

**Result:**

In healthy individuals, TLR9 expression on red blood cell surfaces averaged 8.81%. However, the average expression of TLR9 on red blood cell surfaces in patients with bacterial infection was 5.45%, which was lower than that in healthy people (*p* < 0.001). Notably, both healthy individuals and infected patients exhibited mtDNA binding to red blood cell surfaces, with patients demonstrating a higher mtDNA copy number compared to healthy controls (*p* < 0.001). Moreover, within the infected group, the copy numbers of mtDNA bound by red blood cells positively correlated with patient CRP concentrations (*R*^2^ = 0.715, *p* < 0.001), indicative of an association between mtDNA copy numbers bound to red blood cell surfaces and infection severity.

**Conclusion:**

The elevation of erythrocyte-bound mtDNA during infection, coupled with its correlation with infection severity, suggests that monitoring the copy numbers of mtDNA bound to red blood cells via TLR9 could serve as a novel indicator for infection surveillance.

## 1 Introduction

Traditionally, red blood cells have been viewed solely as carriers of respiratory gases due to their lack of nuclei and organelles. However, their sheer abundance and ability to traverse organs and tissues have long hinted at a more complex role. Despite their simplicity, red blood cells facilitate crucial signal transmission between different tissues, organs, and immune components (1). In recent years, the immune function of red blood cells has become a hot spot in the field. Both mature red blood cells and nucleated red blood cells have been reported to have their own immune potential, which can participate in the regulation of innate and adaptive immune systems (2–5). The evidence that these red blood cells participate in immune regulation has opened up a new field of immunology.

Mitochondrial DNA (mtDNA), as a damage-associated molecular pattern, has the potential to incite inflammatory reactions and contribute to organ damage. Serving as a critical activator of inflammation and the innate immune system, it represents an endogenous molecule capable of triggering toll-like receptor 9 (TLR9) activation (6, 7). When cells are stressed or damaged, mtDNA is excreted from mitochondria and enters the surrounding environment of cells. The combination between mtDNA and TLR9 can trigger inflammation and autoimmune diseases (8).

Toll-like receptors (TLRs) are a family of evolutionarily conserved pattern recognition receptors (PRRs) capable of eliciting the secretion of inflammatory cytokines and stimulating the generation of antigen-specific immune responses within the body (9–11). Hemmi et al. (12) found that TLR9 that can recognize and bind bacterial DNA. TLR9 is typically situated within cells and serves a crucial function in discerning pathogenicity and self-DNA in chronic inflammation and immune complexes. DNA containing CpG motifs (unmethylated cytosine-guanine nucleotide sequences) can be identified, including DNA fragments harboring CpG sequences found in bacteria, viruses, and fungi, or DNA fragments containing CpG sequences themselves, such as mtDNA (6, 13). For a significant duration, TLR9 was presumed to be exclusively expressed in certain immune cells, such as plasmacytoid dendritic cells (pDCs), monocytes, macrophages, activated T cells, and memory B cells (14). However, a few studies have suggested the expression of TLR9 in red blood cells. Recent investigations not only confirmed the presence of TLR9 in erythrocytes but also identified its expression on the erythrocyte membrane. Furthermore, these studies also found that red blood cells combined with different amounts of mtDNA through TLR9 play different immunomodulatory roles, which can promote the maintenance of homeostasis and activate innate immunity (15, 16). This provides preliminary evidence for red blood cells to participate in immune regulation through TLR9 binding to mtDNA. In this study, we want to further explore whether the copy number of mtDNA bound by red blood cells through TLR9 can become a new indicator of infection monitoring.

## 2 Methods

### 2.1 Research object

64 EDTA-K2 anticoagulant whole blood samples were randomly collected from infected patients at Deyang People’s Hospital between June and October 2023 (aged 18–95,40 males and 24 females). Inclusion criteria comprised bacterial infection, with laboratory examination indicating plasma C-reactive protein (CRP) levels exceeding two standard deviations of normal (CRP concentration ranges from 24.16 mg/L to 228.90 mg/L, with an average of 96.71 mg/L). This study was approved by the Ethics Committee of Deyang People’s Hospital (approval number: 2022–04-066-K01).

100 anticoagulated whole blood samples were randomly collected from unpaid blood donors at the Deyang Central Blood Station.

### 2.2 Separation of red blood cells

The anticoagulated whole blood samples were diluted with PBS at a 1:1 ratio. Subsequently, anticoagulant-treated Ficoll-Paque liquid (Cytiva, USA), equal in volume to the diluted blood, was slowly added to the surface. The mixture was then centrifuged at room temperature for 35 min at 400 g with a slow acceleration and deceleration. Following centrifugation, the sample was fractionated into four layers. The top three layers were meticulously aspirated and discarded using a pipette, while the bottom layer, consisting of red blood cells, was retained. Using anti-CD235a (erythrocyte surface specific antigen) antibody and anti-CD45 (leukocyte specific antibody) antibody, the separated red blood cells were detected by flow cytometry, and the purity of the extracted red blood cells was determined to eliminate leukocyte pollution.

### 2.3 TLR9 detection

Following fractionation, 100 μL of red blood cell suspension diluted with PBS (containing not less than 10^5^ red blood cells) was added. Subsequently, 1 μL of biotin Anti-TLR9 antibody (Abcam, UK) was added, and the mixture was incubated in the dark on ice for 30 min. Afterward, 1 mL of stain buffer was added to wash the cells. Upon discarding the supernatant, 1 μL of FITC-conjugated affinipure goat anti-mouse IgG antibody (Proteintech, USA) was added, and the mixture was further incubated for 30 min in the dark on ice. Following this incubation period, another 1 mL of stain buffer was added to wash the cells. Subsequently, the supernatant was discarded, and the cells were resuspended in 300–400 μL of stain buffer for flow cytometry analysis.

### 2.4 Co-culture

1 mL of plasma from patients with the same ABO and Rh blood types was co-cultured with 100ul of healthy red blood cells (no less than 10^9^ red blood cells) at 4°C for 24 h, during which strict aseptic operation was carried out. After that, detected the expression of TLR9 on the surface of red blood cells before and after culture.

### 2.5 DNA extraction

At room temperature, DNA was extracted from 10^8^ red blood cells by DNA extraction kit (Qiagen, Germany) and stored at −20°C.

### 2.6 qPCR detection

Prepare a 96-well plate and sequentially add 10 μL of MIX, 2 μL of DNA sample, 7.2 μL of ultrapure water, 0.4 μL of forward primer, and 0.4 μL of reverse primer to each well, the primer sequences are shown in Table 1. After adding the components, gently mix the plate and seal it with a plate sealing film. Centrifuge the plate to precipitate the liquid. Subsequently, perform real-time fluorescence quantitative PCR detection using a computer and amplify according to the procedures outlined in Table 2.

**Table 1:** qPCR primer.

Gene	Forword primer 5′–3′	Reverse primer 5′–3′
Human mtDNA	ACGACCTCGATGTTGGATC	GCTCTGCCATCTTAACAAACC

**Table 2:** qPCR reaction system.

Procedure	Temperature/°C	Time/S	Cyclic number
Predeformation	94	30	1
Deformed	94	5	40
Anneal	60	15	40
Extend	72	10	40
Dissolution curve	65	5	1
	95	50	1

### 2.7 Statistical method

The data were analyzed using SPSS version 27.0 software, employing univariate logistic analysis. T-test analysis was conducted using GraphPad Prism 9 software, with statistical significance set at *p* < 0.05.

## 3 Results

### 3.1 TLR9 is expressed on the surface of red blood cells

**Figure 1:**
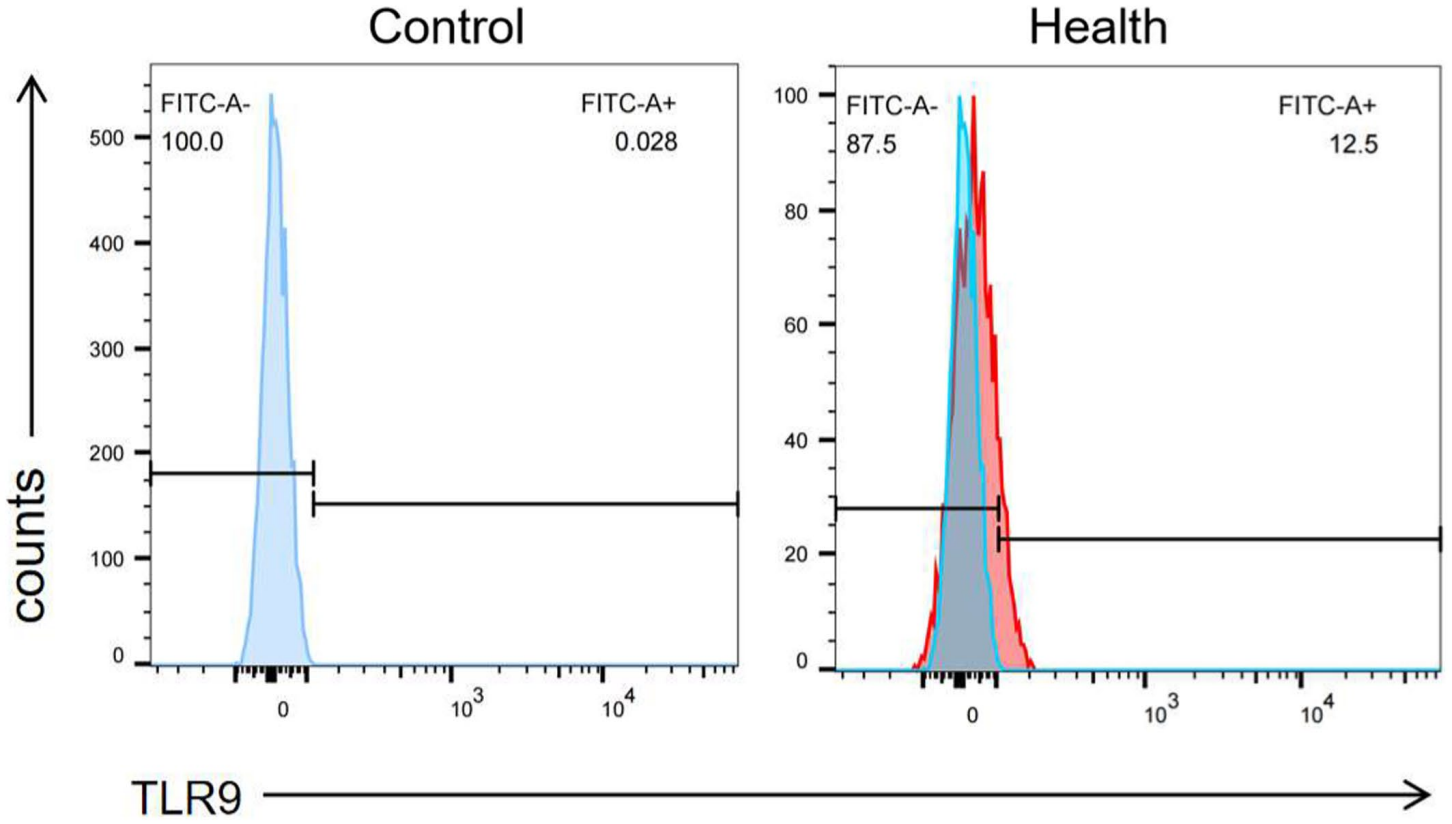
The expression of TLR9 on the surface of healthy human erythrocytes was detected by flow cytometry and the negative control was made.

**Figure 2:**
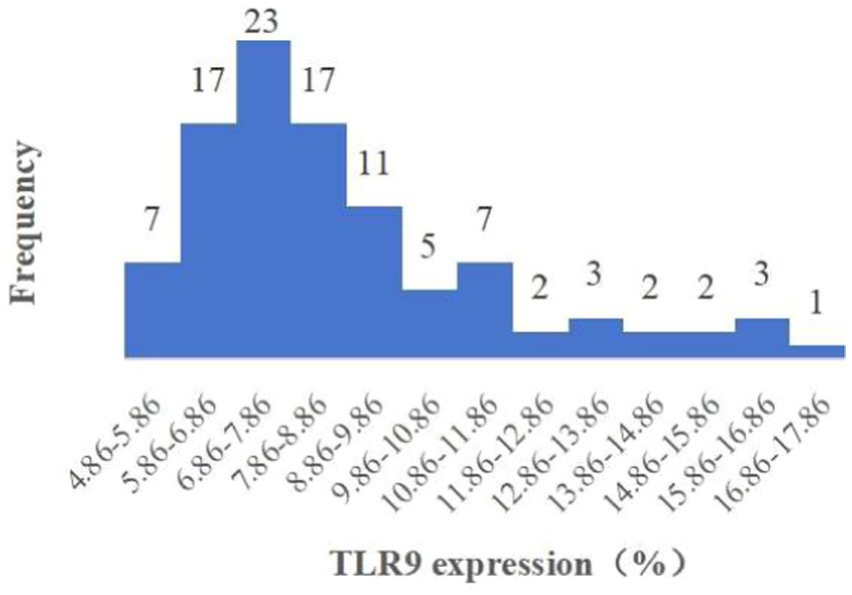
Histogram of expression of TLR9 on erythrocyte surface of 100 healthy people.

When assessing TLR9 expression on the surface of red blood cells from 100 healthy individuals using flow cytometry with a specific membrane epitope antibody (16), we observed TLR9 expression on all samples (Figure 1). The average expression level was determined to be 8.81% (Figure 2). These findings confirm the presence of TLR9 not only within red blood cells but also on their surface.

### 3.2 Differential expression of TLR9 on the surface of red blood cells between healthy people and patients with bacterial infection

**Figure 3:**
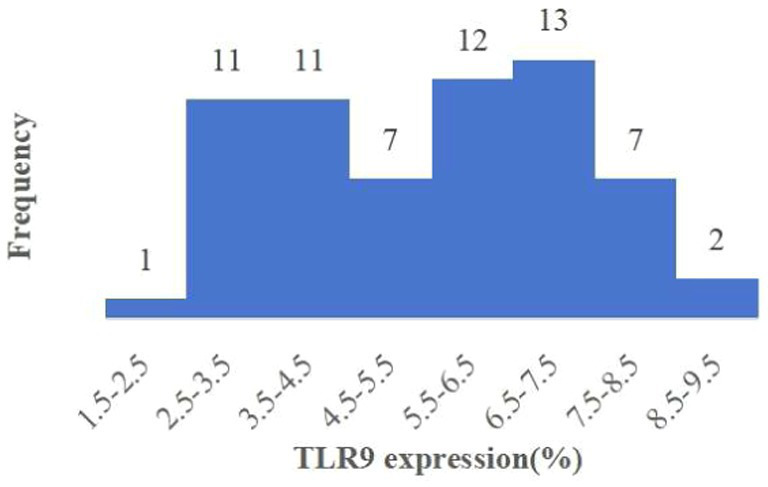
Histogram of expression of TLR9 on erythrocyte surface of 64 patients.

**Figure 4:**
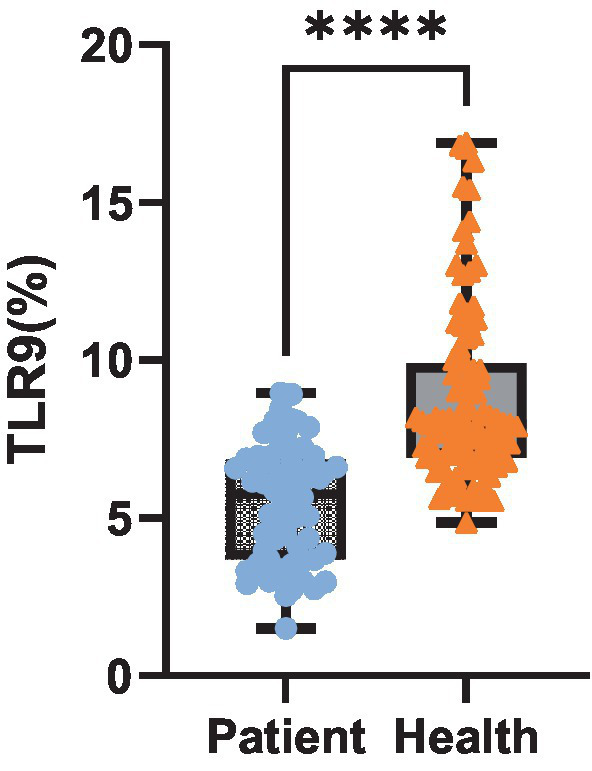
Expression level of TLR9. Expression of TLR9 on erythrocyte surface in 64 patients and 100 healthy peopel. ****p < 0.001.

Upon analyzing TLR9 expression in 64 patients with bacterial infection, we observed the average expression of TLR9 on the surface of patients’ red blood cells was 5.45% (Figure 3), which was less than that of healthy people (*p* < 0.001) (Figure 4). These results confirm a notable difference in TLR9 expression on the surface of red blood cells between patients with bacterial infection and healthy individuals.

### 3.3 During bacterial infection, red blood cells bind to mtDNA through TLR9

**Figure 5:**
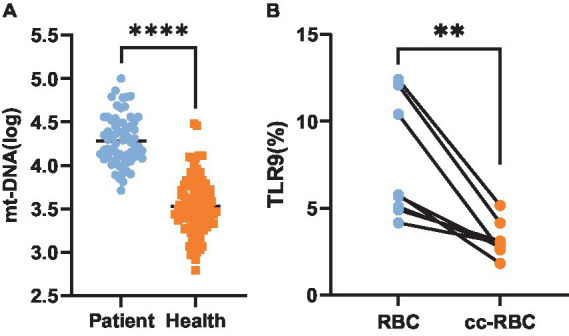
**(A)** MtDNA copy number. Copy number of mtDNA bound to red blood cells in 64 patients and 100 healthy people. **(B)** Expression level of TLR9. The expression amount of TLR9 on the surface of healthy human erythrocytes and the expression amount of TLR9 on the surface of healthy human erythrocytes after 24 h of co-culture with the plasma of patients with bacterial infection, *n* = 8. **p < 0.01; ****p < 0.001.

It has been established that TLR9 is expressed on the surface of red blood cells, suggesting the presence of potential mtDNA binding sites on these cells^[13, 16]^. Hence, we aimed to investigate whether red blood cells can bind mtDNA during infection. Quantitative PCR was employed to assess the red blood cells of 100 healthy individuals and 64 patients with bacterial infection. The results showed that the average copy number of mtDNA bound to the surface of healthy people’s erythrocytes was 3,548 ± 2.08/ul, and that of patients’ erythrocytes was 19,952 ± 1.9/ul. The results revealed a significant increase in the copy number of mtDNA bound to red blood cells in patients compared to the healthy control group (*p* < 0.001) (Figure 5A).

We speculated whether the disparity in TLR9 expression on the surface of red blood cells between healthy individuals and patients with bacterial infection could be attributed to increased mtDNA binding, potentially obstructing the detection sites of TLR9. To explore this hypothesis, we co-cultured plasma from patients with bacterial infection with healthy red blood cells for 24 h and subsequently assessed the expression of TLR9 on the red blood cell surface. Remarkably, compared to healthy red blood cells before co-cultured, the expression of TLR9 on co-cultured red blood cells decreased significantly (*p* < 0.01) (Figure 5B). These findings support our hypothesis that mtDNA may obscure the detection sites of TLR9, leading to reduced TLR9 expression on the surface of red blood cells in patients with bacterial infection compared to healthy individuals.

### 3.4 The copy number of mtDNA bound to erythrocytes increased with the increase of disease severity

**Figure 6:**
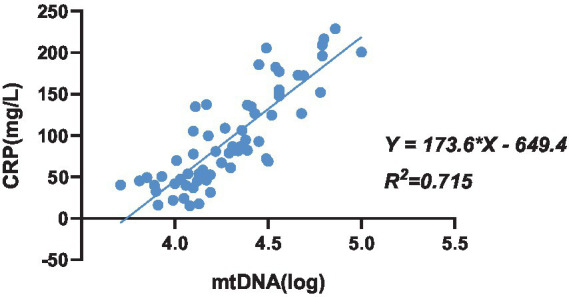
The univariate regression analysis between the copy number of mtDNA bound to erythrocytes and CRP concentration in patients with bacterial infection, n = 64.

C-reactive protein (CRP) serves as an inflammatory marker, characterized by a rapid increase in plasma concentration during infection or tissue damage. In this study, CRP concentration was utilized to gauge the severity of infection. Univariate regression analysis was conducted to assess the relationship between the copy number of mtDNA in red blood cells and CRP concentration in 64 patients with bacterial infection. The analysis revealed a positive correlation between the copy number of mtDNA bound to red blood cells and CRP concentration (*R*^2^ = 0.715; *p* < 0.001) (Figure 6).

## 4 Discussion

Red blood cells have been regarded as lacking immune functions despite being the most abundant cells in the blood. However, their sheer abundance and ability to traverse the nooks and crannies of organs and tissues enable them to facilitate signal transmission between different tissues, organs, and immune components (1). With advancements in research, red blood cells have emerged as more than mere oxygen and carbon dioxide carriers, they are increasingly recognized another important function of red blood cells in mammals, that is, immune regulation function, and red blood cells can perform their immune function in many ways (17). Numerous domestic and international studies have validated the presence of numerous immune-related molecules on red blood cells, underscoring their role in complex natural immunity processes such as antigen recognition, adhesion, killing, and clearance of circulating immune complexes (CICs) (18). In addition, red blood cells also participate in immune response through other channels. For example, Sennikov et al. (19) found that nucleated red blood cells in human bone marrow are capable of producing a variety of cytokines, including interleukin (IL)-1β, IL-2, IL-4, IL-6, interferon (IFN)-*γ*, among others, actively engaging in immune regulation. In patients infected with COVID-19, ROS originating from red blood cells have been implicated in the exacerbation of endothelial dysfunction, potentially leading to multiple organ failure and microvascular thrombosis (20). Erythrocyte Duffy antigen, chemokine receptor (DARC), can bind chemokines and play an important role in anti-tumor and inflammatory immunity (21); Red blood cells mediate plasmodium and SARS-CoV-2, norovirus, dengue fever and other viruses through glycoprotein on the surface (22–25); Erythrocyte surface complement receptor 1 (CR1) is also involved in the immune regulation of patients with mycoplasma and COVID-19 (26, 27). However, it is still unclear what role red blood cells play in monitoring infectious diseases.

TLRs recognize specific microbial structures. TLR9 is stimulated by unmethylated cytosine guanine nucleotide sequences (CpGs) in DNA fragments of bacteria, viruses and fungi, as well as synthetic oligonucleotides or their own mitochondrial DNA (10, 13). For a considerable period, TLR9 has conventionally been viewed as a pattern recognition receptor predominantly expressed on the cell membrane and limited to specific immune cell populations (11). A study by Meghan (15) and others in 2018 found that TLR9 was also expressed in red blood cells, which broke the traditional cognition of TLR9 expression. In 2021, Lam et al. (16) found that TLR9 was expressed not only in red blood cells, but also on the surface of red blood cells by flow cytometry and confocal microscope. In this study, red blood cells of healthy people and patients with bacterial infection were detected by using specific membrane epitope antibodies. It was found that a small amount of TLR9 was generally expressed on the surface of red blood cells of healthy people, with an average expression of 8.81%. However, the average expression of TLR9 on red blood cell surfaces in patients with bacterial infection was 5.45%, which was lower than that in healthy people (*p* < 0.001). This is contrary to the research results of Lam et al. (16), who found that the surface TLR9 of red blood cells in septicemia patients was increased compared with that of healthy donors. We consider that there may be the following reasons: A. The severity of the disease is different: Lam et al. (16) studied critically ill patients with sepsis. Sepsis is a very serious inflammatory disease with systemic inflammation and immune response. They consider that excessive mtDNA will affect red blood cells, which may lead to the increase of detectable TLR9 epitopes on their surface. However, our research team is targeted at patients with common infection, and the severity of the disease is far less than that of severe sepsis, so the amount of mtDNA bound to TLR9 is small, which is not enough to affect red blood cells and TLR9 on their surfaces. From our research results, or because the combination of mtDNA and TLR9 masked the detection epitope of TLR9, the detectable TLR9 on the surface of patients’ red blood cells decreased. Therefore, the different disease severity of the subjects may be one of the reasons for the different results. B. Inconsistent number of subjects: 15 healthy people and 19 septicemia patients were included in the study of Lam et al. (16). Our study included 100 healthy people and 64 infected patients. The difference in the number of research objects may also be a reason for the difference in statistical results.

As a damage-associated molecular pattern, mtDNA can trigger inflammatory reactions and organ damage, serving as a crucial activator of inflammation and the innate immune system, and can be recognized by TLR9 (6, 7). Meghan et al. (15) found that under normal physiological conditions, most of mtDNA exists on red blood cells instead of plasma, and red blood cells bind to mtDNA containing CpG sequence in a concentration-dependent manner. And their research also found that TLR9 positive cells obtained more CpG-DNA than TLR9 negative cells. In this study, quantitative PCR analysis was conducted on samples from 100 healthy individuals and 64 patients with bacterial infection, the results indicated a significant increase in the number of mtDNA copies bound to red blood cells in patients compared to the healthy control group (*p* < 0.001). This finding provides insight into the potential explanation for the observed lower expression of TLR9 on the surface of red blood cells in patients with bacterial infection compared to healthy individuals: perhaps due to the increased binding of mtDNA to the surface of red blood cells in infected patients, which may obscure the detection sites of TLR9, resulting in reduced TLR9 expression on the surface of red blood cells in patients with bacterial infection. We detected the expression of TLR9 on the surface of red blood cells after co-culturing the plasma of patients with bacterial infection with healthy red blood cells for 24 h. It was found that the expression of TLR9 on the surface of red blood cells after co-cultured was significantly lower than that of healthy red blood cells before co-cultured. This may confirm our guess that a large amount of mtDNA in infected patients binds to TLR9, which obscures the detection site of TLR9. We just put forward a guess and verified it, or there are many other reasons that lead to less TLR9 expression on the surface of patients’ red blood cells than healthy people.

Lam et al. (16) have confirmed that TLR9 combined with mtDNA can indeed trigger innate immunity during infection. However, whether red blood cells can monitor diseases via TLR9 and mtDNA during bacterial infection remains unclear. Our study has confirmed an increase in the copy number of mtDNA bound to red blood cells during infection, though its relationship with infection severity requires further investigation. CRP an inflammatory marker, exhibits a sharp increase in plasma levels during infection or tissue damage. In this study, CRP concentration serves as an indicator of infection severity. Through univariate regression analysis of CRP concentration and the copy number of mtDNA bound to red blood cells in 64 bacterial infection patients, we observed a positive correlation. Specifically, the copy number of mtDNA copies bound to red blood cells via TLR9 positively correlated with patients’ CRP concentration (*R*^2^ = 0.715; *p* < 0.001).

However, there are some limitations in this study: (a) Red blood cells were not co-cultured with the plasma of healthy donors for comparison, so as to rule out the possibility that other molecules (such as protein) interfere with the binding of antibodies to TLR9. (b) No linear regression was performed with healthy donors to rule out bias. These are all related to further in-depth study in the future.

In a word, this study confirmed that TLR9 is expressed on the surface of red blood cells, serving as a critical immunosensor capable of binding to free mtDNA within cells. Furthermore, the copy munber of mtDNA bound by red blood cells via surface TLR9 increased during bacterial infection, correlating positively with the severity of the infection. Hence, detecting the copy number of mtDNA bound by red blood cells through TLR9 may emerge as a novel indicator for monitoring infection within the body.

## Data Availability

The original contributions presented in the study are included in the article/Supplementary material, further inquiries can be directed to the corresponding authors.
