# Supplementary material for: Preliminary study on the participation of TLR9 on erythrocyte surface combined with mtDNA in the monitoring of infectious diseases
Source: Front Med (Lausanne). 2025 Feb 19;11:1498627. doi: 10.3389/fmed.2024.1498627 (PMC11879980; doi:10.3389/fmed.2024.1498627)
Supplement: Supplementary file 1 [file Data_Sheet_1.pdf]

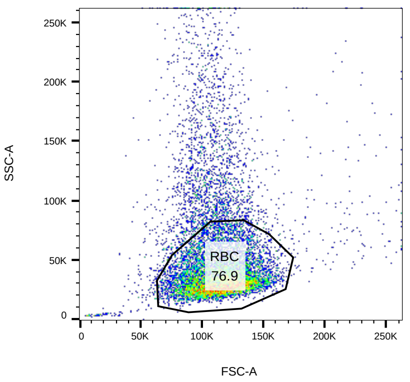

CD235+CD45_BLANK01.fcs
Ungated
10000

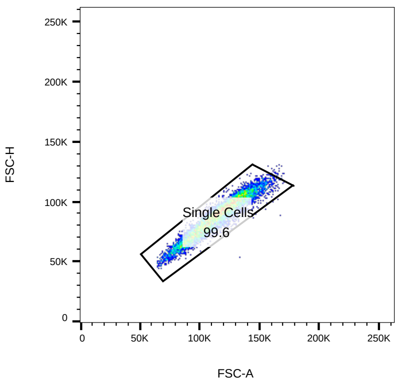

CD235+CD45_BLANK01.fcs
RBC
7692

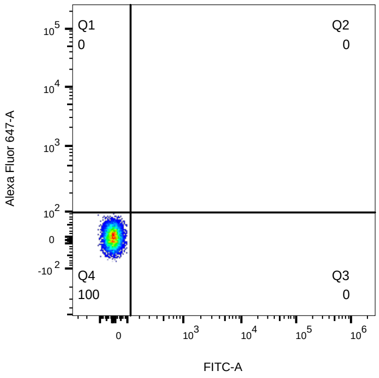

CD235+CD45_BLANK01.fcs
Q4: FITC-A- , Alexa Fluor 647-A-
7660

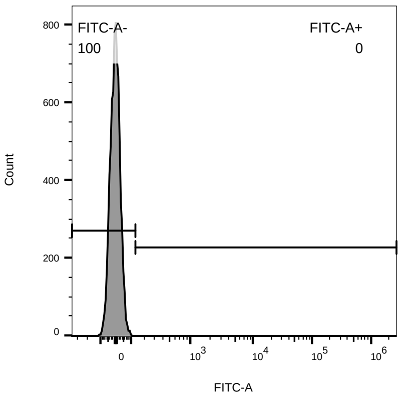

CD235+CD45_BLANK01.fcs
Single Cells
7665

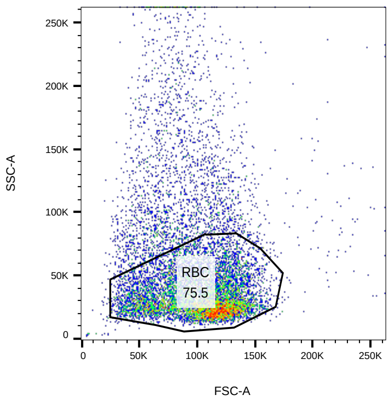

CD235+CD45 -01.fcs
Ungated
10000

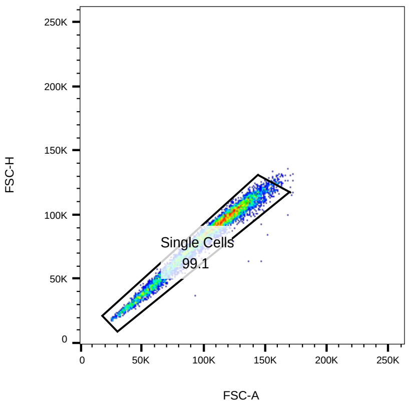

CD235+CD45 -01.fcs
RBC
7548

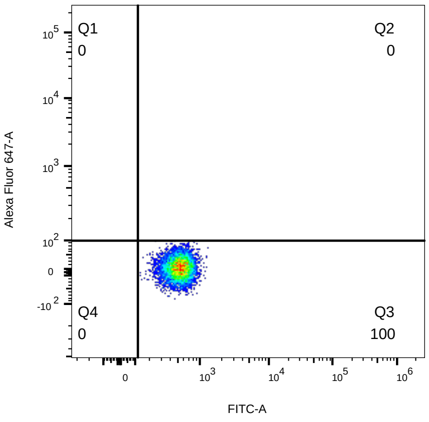

CD235+CD45 -01.fcs
Q3: FITC-A+ , Alexa Fluor 647-A-
7476

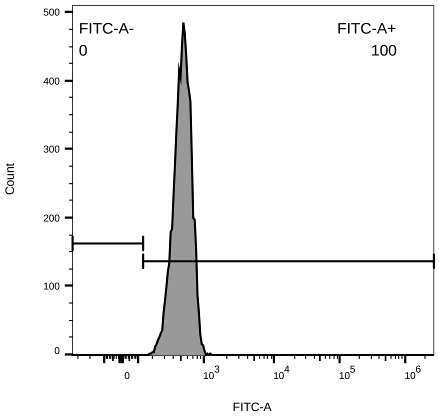

CD235+CD45 -01.fcs
Single Cells
7479

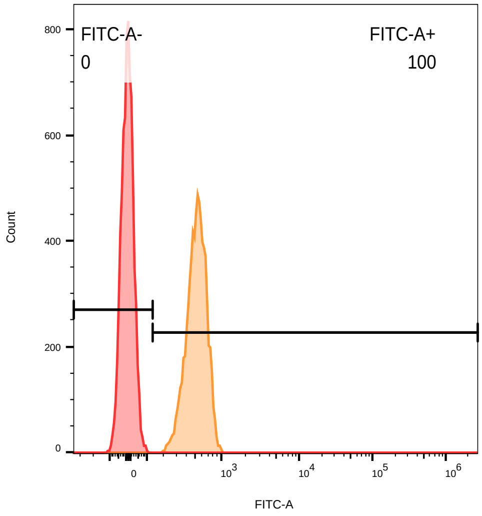

	Sample Name	Subset Name	Count
(red)	CD235+CD45_BLANK01.fcs	Single Cells	7665
(orange)	CD235+CD45 -01.fcs	Single Cells	7479
